# Are Counterattacks More Effective than Positional Attacks in Soccer? A Comparative Analysis of Influencing Factors

**DOI:** 10.5114/jhk/202435

**Published:** 2025-09-23

**Authors:** Pablo Prieto-González, Víctor Martín, Rui Marcelino, Alejandro Sal-de-Rellán

**Affiliations:** 1 Sport Sciences and Diagnostics Research Group, College of Humanities and Sciences, Prince Sultan University, Riyadh, Saudi Arabia.; 2 Faculty of Health Sciences, Universidad Isabel I de Castilla, Burgos, Spain.; 3 Research Center in Sports Sciences, Health Sciences and Human Development, CIDESD, Elite Research Community, Maia, Portugal.; 4 Sport Science Department, University of Maia, Maia, Portugal.; 5 FPF Academy, Portuguese Football Federation, Oeiras, Portugal.; 6 Department of Education and Educational Innovation, Faculty of Law, Education and Humanities, Universidad Europea de Madrid, Madrid, Spain.

**Keywords:** tactical precision, offensive strategies, team performance metrics, league-specific analysis, central-zone efficiency

## Abstract

This study aimed to: (1) compare the efficiency of positional attacks and counterattacks in La Liga, the Premier League, and Serie A, considering team rankings (top, intermediate, and bottom tiers); and (2) identify key factors that influenced the success of both types of attack. A quantitative, observational study adhering to STROBE guidelines was conducted. Data from five seasons (2017–2022) were collected from INSTAT, covering 5,700 matches across the three selected leagues. The analysis included 84 teams. Of the 115 team variables available, 35 independent and two dependent variables—efficiency in positional attacks and counterattacks—were selected. These variables included team performance metrics and tactical indicators. Counterattacks were more efficient than positional attacks across the three leagues, and top teams performed better than intermediate and bottom teams. Serie A showed the highest efficiency. In La Liga and the Premier League, positional attack efficiency was linked to right flank attacks, while counterattacks relied on central-zone efficiency. Serie A showed similar patterns, with the right flank contributing most to positional attack efficiency and central-zone efficiency being the strongest predictor of counterattack efficiency. The superiority of counterattacks over positional attacks underscores the importance of tactical precision and situational execution. Top teams excelled in both strategies, emphasizing the need for efficiency. The findings highlight the significance of adapting strategies to each league’s unique trends. Coaches can leverage these insights to refine their approach, focusing on fast transitions, possession play, and wing play to enhance attacking efficiency and overall team performance.

## Introduction

Soccer is one of the most popular sports worldwide, characterized by its constant dynamic nature and tactical diversity (Marcelino et al., 2020). Within this context, a team's success depends largely on its ability to adapt to different game situations and employ effective offensive strategies (Hewitt et al., 2016). Two of the most popular offensive strategies are positional attacks and counterattacks (Lago-Peñas et al., 2017; Sarmento et al., 2018). The positional attack aims to progressively build up game through ball possession, seeking to disorganize the opposing defense through players' constant movement and space creation (da Costa et al., 2009). However, counterattacks are characterized by their speed and ability to surprise. Teams generally execute counterattacks when they regain possession of the ball and immediately take advantage of defensive disorganization to attack quickly (Hewitt et al., 2016).

The evolution of tactical approaches in different leagues, each characterized by unique styles of play and match rhythms, reflects the complexity of soccer (Sarmento et al., 2013). For example, the English Premier League (EPL) is famous for its fast and direct style of play, with a higher tempo, frequent duels and a more aggressive attitude (Dellal et al., 2011). However, the best-ranked clubs in this league have adopted a possession-based style in recent years marking an important tactical evolution (Bradley et al., 2016). On the other hand, in both the Spanish League and the Italian League, tactical and contextual factors play an important role in collective performance (Sarmento et al., 2013). In the Spanish League, the game is more elaborate, allowing teams to successfully interpret various moments, which is why they have an exceptional reputation for their quality of play (Mitrotasios et al., 2019). However, Italian soccer is more focused on the defensive tactical aspects, causing the players to be more “tied-up” and have less freedom to play (Papadopoulos et al., 2021; Sarmento et al., 2013). The analysis of players’ movements and game dynamics in the major leagues has been the subject of several research studies (Barthelemy et al., 2024; Li and Zhao, 2021; Mitrotasios et al., 2019; Sarmento et al., 2013). Counterattacks proved to be the most effective strategy compared to positional attacks (Lago-Ballesteros et al., 2012; Sarmento et al., 2018; Tenga et al., 2010a), especially when facing a disorganized defense (Lago-Ballesteros et al., 2012; Sarmento et al., 2018; Tenga, et al., 2010a). It has also been observed that quick attacks were more effective than positional attacks in achieving offensive penetration, but not in creating scoring opportunities. In contrast, direct attacks were less effective than positional attacks in achieving offensive performance (González-Ródenas et al., 2021). In addition to the game analysis, scientific literature has demonstrated that the top-ranked teams exhibit a more combinative style of play (Gómez et al., 2018), a higher percentage of ball possession (Bradley et al., 2014), perform a greater number of offensive transitions (Casal et al., 2021), use less direct play (González-Ródenas et al., 2021), and are more proactive in the game (Lopez-Valenciano et al., 2022).

However, accurately assessing playing styles and their effectiveness requires a more nuanced analysis that considers the interaction of multiple tactical variables, team strategies, and contextual factors, such as match dynamics and opponent quality. This study not only addresses gaps in the understanding of positional and counterattack effectiveness in elite European soccer—specifically in La Liga, the English Premier League, and Serie A—but also provides valuable insights for coaches, analysts, and teams aiming to optimize their offensive strategies based on league-specific trends and tactical influences.

Characterizing playing styles and their effectiveness requires a more nuanced analysis considering the interaction of various tactical variables, team strategies, and contextual influences, such as the match's state and the opponent's quality. The relevance of conducting this study lies in the need to address gaps in the understanding of the effectiveness of positional attacks and counterattacks in elite European football, specifically in La Liga, the English Premier League, and Serie A. By analyzing the distinct tactical characteristics of each league, the findings of this study could provide valuable insights to help coaches and analysts optimize offensive strategies in diverse competitive contexts.

With the help of a robust dataset from the INSTAT platform, this study aimed to: (1) compare the effectiveness of positional attacks and counterattacks across different leagues and team classifications (top, intermediate, and bottom tiers within each league); and (2) find the key factors that affected the outcomes of these attacking strategies in a range of match situations. It was hypothesized that counterattacks would produce higher success rates than positional attacks, particularly when faced with less organized defensive structures (Tenga et al., 2010b). Furthermore, the effectiveness of these attacking approaches was expected to show considerable variability between different leagues, team classifications and specific game contexts (Prieto-González et al., 2024; Sarmento et al., 2013).

## Methods

### Study Design

A quantitative, observational research was conducted. The study received ethical approval from the institutional review board at the Prince Sultan University, Saudi Arabia (approval code: PSU IRB-2024-07-5336; approval date: 21 July 2024), ensuring compliance with ethical standards. It was conducted in accordance with the principles outlined in the Declaration of Helsinki and followed the guidelines set forth by the STROBE (Strengthening the Reporting of Observational Studies in Epidemiology) checklist.

### Setting and Participants

To ensure a comprehensive analysis, the study included all professional teams from the first divisions of the Spanish La Liga, the English Premier League, and the Italian Serie A, the three best world leagues (UEFA, 2024). The dataset spanned five seasons, from 2017–2018 to 2021–2022, and was sourced from the INSTAT platform. It covered all 20 teams from these top-tier leagues across each season, totaling 5,700 matches analyzed. Due to the relegation and promotion system of the three leagues analyzed, 84 teams were included. Of these, 28 belonged to the Spanish La Liga, 28 to the English Premier League, and 28 to the Italian Serie A.

### Variables

The INSTAT database contains a total of 115 team variables. From the available set of variables, the four main researchers of this study independently and blindly conducted a selection process to identify those relevant to the present study—specifically, those that could influence the effectiveness of positional attacks and counterattacks. Thus, only the variables selected by all four main researchers were included in the study. In this way, the study included two dependent variables: percentage efficiency for positional attacks and percentage efficiency for counterattacks. The independent variables included were the following 35, shown in Table 1.

**Table 1:** Independent variables included in the study. Annual averages from the 38 matches of each season are shown.

	La Liga	English Premier League	Serie A
Accurate Crosses	131.11 ± 34.78	121.06 ± 24.25	138.68 ± 32.24
Accurate Key Passes	113.5 ± 34.98	116.64 ± 38.03	112.13 ± 33.33
Accurate Passes	14706.82 ± 3545.24	15507.97 ± 3819.68	15733.72 ± 2809.11
Attacks in the Center	770.14 ± 105.45	755.43 ± 110.38	801.27 ± 89.65
Attacks on the Left Flank	1017.57 ± 117.80	1021.92 ± 102.42	1005.13 ± 123.77
Attacks on the Right Flank	1046.61 ± 109.58	1006.08 ± 112.45	1006.03 ± 120.27
Attacks with Shots in the Center	94.05 ± 28.16	107.96 ± 34.05	123.6 ± 32.67
Attacks with Shots on the Left Flank	92.77 ± 23.80	98.75 ± 25.30	104.9 ± 29.66
Attacks with Shots on the Right Flank	90.51 ± 20.70	91.48 ± 23.79	101.24 ± 25.96
Average Duration of Ball Possession in Seconds	15.03 ± 2.67	15.9 ± 2.89	15.83 ± 2.11
Chances	191.65 ± 48.26	203.33 ± 53.51	205.74 ± 52.87
Counterattacks	496.73 ± 65.17	494.53 ± 61.23	489.68 ± 67.34
Counterattacks with a Shot	72.3 ± 16.63	76.47 ± 16.41	86.07 ± 19.67
Crosses	507.22 ± 101.78	481.38 ± 74.61	507.65 ± 92.21
Dribbles	942.19 ± 151.72	936.38 ± 127.44	934.22 ± 130.22
Efficiency for Attacks through the Central Zone in Percentage	12.1 ± 2.72	14.16 ± 3.35	15.34 ± 3.37
Efficiency for Attacks through the Left Flank in Percentage	9.11 ± 1.99	9.6 ± 2	10.41 ± 2.27
Efficiency for Attacks through the Right Flank in Percentage	8.63 ± 1.85	9.08 ± 2.03	10.12 ± 2.28
Entrances to the Final Third	1471.53 ± 175.68	1492.3 ± 208.46	1508.68 ± 189.07
Entrances to the Opposition Half	2284.26 ± 188.77	2254.62 ± 211.71	2297.76 ± 212.75
Entrances to the Penalty Box	546.85 ± 111.68	583.41 ± 133.11	585.09 ± 107.95
Key Passes	262.45 ± 84.86	265.13 ± 91.77	248.15 ± 66.82
Passes	17820.75 ± 3415.51	18667.21 ± 3812.68	18657.89 ± 2779.18
Percentage of Accurate Crosses	25.66 ± 2.97	25.09 ± 2.98	27.25 ± 3.63
Percentage of Accurate Passes	81.83 ± 4.07	82.44 ± 3.47	84.03 ± 2.71
Percentage of Ball Possession	49.83 ± 5.77	49.8 ± 7.00	49.93 ± 4.98
Percentage of Chances	25.23 ± 3.88	25.49 ± 4.24	26.38 ± 4.06
Percentage of Shots on Target	38.98 ± 3.52	38.86 ± 2.92	37.75 ± 3.09
Percentage of Successful Dribbles	56.59 ± 3.68	56.78 ± 3.51	57.68 ± 3.31
Positional Attacks	2337.59 ± 165.18	2288.9 ± 195.06	2322.75 ± 179.50
Positional Attacks with Shots	209.09 ± 50.28	227.01 ± 64.57	247.71 ± 62.35
Quantity of Ball Possessions	3931.2 ± 181.64	3852.1 ± 189.58	3901.36 ± 208.16
Shots	412.44 ± 69.81	441.45 ± 88.98	476.21 ± 92.36
Shots on Target	161.32 ± 36.22	172.61 ± 41.34	180.38 ± 41.49
Successful Dribbles	534.86 ± 105.17	533.1 ± 86.14	539.28 ± 84.85

### Bias

To address potential sources of bias, the intraclass correlation coefficient (ICC) was used to assess the consistency of each of the 35 independent variables and the two dependent variables across the three leagues included, over the five analyzed seasons. The high ICC values obtained were for the Spanish La Liga (single measures ≥ 0.887 and average measures ≥ 0.963), the English Premier League (single measures ≥ 0.896 and average measures ≥ 0.977), and the Italian Serie A (single measures ≥ 0.891 and average measures ≥ 0.954). These values indicated solid reliability of the data for the three analyzed leagues.

### Statistical Analysis

Data are presented as mean ± standard deviation (SD). Assumptions of normality and homogeneity of variances were verified to ensure the validity of our ANOVA findings. A three-way ANOVA (3 x 3 x 2) was conducted to investigate the interaction effects of the League (Premier League, La Liga, Serie A), team level (“top teams”, “intermediate teams”, and “bottom teams”), and the type of attack (positional attacks, counterattacks) on the efficiency of attacks. To classify teams by the competitive level, a two-step cluster analysis was performed using the log-likelihood distance measure and the Schwarz’s Bayesian criterion (Marcelino et al., 2011). The number of clusters was fixed at three, with variables including points obtained per season (where each win = 2 points and each loss = 1 point), the ratio of total points won and lost, total goals scored and conceded, and the percentage of points won. Based on our two-step cluster analysis, the first cluster, labeled “Top teams”, included the seven teams with the highest points obtained per season. The “Intermediate teams” cluster comprised teams ranking 8^th^ to 13^th^, while the “Bottom teams” cluster included the seven teams with the lowest points obtained per season. This approach was useful to assess differences in attack effectiveness and explore interaction effects between the attack type, the team level, and the league. Post hoc tests were employed to identify specific differences among leagues, the team level, and types of attacks where significant effects were observed. Effect sizes were calculated using the η^2^ parameter and interpreted as follows: 0.2 indicating a small effect, 0.5 indicating a medium effect, and 0.8 indicating a large effect (Lakens, 2013). A regression analysis was performed to identify factors influencing the effectiveness of positional attacks and counterattacks across the three analyzed leagues. Variable selection techniques were employed to manage the large number of independent variables and prevent overfitting. Scatter plots were examined to determine whether a linear or nonlinear regression model was appropriate, with multiple linear regression providing the best fit. The independence of residuals was assessed using the Durbin-Watson statistic, with values between 1.5 and 2.5 considered acceptable for indicating independence. To identify substantial correlations, multicollinearity among predictor variables was evaluated using the variance inflation factor and collinearity tolerance, with thresholds of 10 and 0.1. Given the large number of predictor variables, stepwise regression was chosen for the analysis. Additionally, principal component analysis (PCA) was conducted to investigate multicollinearity and reduce data dimensionality. PCA transformed original variables into uncorrelated principal components that captured maximum data variance. The analysis involved standardizing the data, calculating the covariance matrix, calculating eigenvalues and eigenvectors to identify the principal components, selecting those with eigenvalues greater than 1, and projecting the original data onto these components. PCA results confirmed that the selected components effectively captured the data's structure without collinearity issues, validating the suitability of predictor variables for the regression model. All statistical analyses were performed using IBM SPSS Statistics software (Version 26, USA).

## Results

Based on the results from the repeated measures 3 x 3 x 2 ANOVA (Table 2), a main effect of attack was found (F(1,29) = 868.183, p < 0.001, η^2^ = 0.968), indicating that counterattacks were more efficient than positional attacks. A main effect of league was also observed (F(2,58) = 35.566, p < 0.001, η^2^ = 0.551). The average efficiency of both types of attacks (positional and counterattacks) was significantly higher in Serie A compared to the Premier League, and in the Premier League it was significantly higher than in La Liga. The main effect of the team level was also found (F(2,58) = 84.516, p < 0.001, η^2^ = 0.745), indicating that mean efficiency differed significantly across the three levels of teams. Attacks were significantly more efficient among top teams than intermediate teams, and attacks performed by intermediate teams were significantly more efficient than those by teams at the bottom.

**Table 2:** Effectiveness of positional attacks and counterattacks across leagues and team levels.

Attack type, league, and team level	X̅ ± SD	95% Confidence Interval	
		Lower bound	Upper bound
PA_SP, PL & SA	9.77 ± 2.21	5.01	15.84
CA_ SP, PL & SA	15.97 ± 3.78	7.87	29.67
PA & CA_SP	11.78 ± 3.96	5.12	25.78
PA & CA_PL	12.71 ± 4.17	6.05	24.12
PA & CA_SA	14.13 ± 4.68	7.32	29.27
TT_ PA & CA	15.20 ± 4.53	7.03	29.97
MT_ PA & CA	12.24 ± 4.07	6.11	27.01
BT_ PA & CA	11.09 ± 3.38	5.09	20.04
PA_SP	8.91 ± 0.19	8.52	9.27
CA_SP	14.63 ± 0.34	13.99	15.32
PA_PL	9.81 ± 0.22	9.38	10.24
CA_PL	15.62 ± 0.36	14.89	16.34
PA_SA	10.61 ± 0.23	10.17	11.06
CA_SA	17.65 ± 0.38	16.91	18.39
PA_SP_TT	10.28 ± 1.94	7.14	15.13
PA_SP_IT	8.50 ± 1.38	6.21	12.21
PA_SP_BT	7.85 ± 1.24	5.03	10.71
PA_PL_TT	11.91 ± 1.82	8.12	16.32
PA_PL_IT	9.16 ± 1.31	7.32	12.14
PA_PL_BT	8.25 ± 1.22	6.11	10.21
PA_SA_TT	12.65 ± 1.58	10.43	16.34
PA_SA_IT	10.06 ± 1.85	7.12	14.29
PA_SA_BT	9.05 ± 1.49	7.21	13.17
CA_SP_TT	17.17 ± 3.31	11.42	26.24
CA_SP_IT	13.70 ± 2.71	9.16	19.22
CA_SP_BT	12.97 ± 2.35	8.18	18.14
CA_PL_TT	18.51 ± 2.68	14.21	24.19
CA_PL_IT	14.70 ± 3.61	9.14	22.04
CA_PL_BT	13.51 ± 2.50	10.51	18.07
CA_SA_TT	20.65 ± 3.12	13.32	30.15
CA_SA_IT	17.33 ± 3.21	13.15	27.09
CA_SA_BT	14.91 ± 2.29	11.22	20.23

Legend: PA_SP, PL & SA: Positional Attacks in Spanish La Liga, English Premier League, and Italian Serie A; CA_SP, PL & SA: Counterattacks in Spanish La Liga, English Premier League, and Italian Serie A; PA & CA_SP: Positional and Counterattacks in Spanish La Liga; PA & CA_PL: Positional and Counterattacks in English Premier League; PA & CA_SA: Positional and Counterattacks in Italian Serie A; TT_ PA & CA: Top teams – Positional and Counterattacks; MT_ PA & CA: Intermediate teams – Positional and Counterattacks; BT_ PA & CA: Bottom Teams – Positional and Counterattacks; PA_SP: Positional Attacks in Spanish La Liga; CA_SP: Counterattacks in Spanish La Liga; PA_PL: Positional Attacks in English Premier League; CA_PL: Counterattacks in English Premier League; PA_SA: Positional Attacks in Italian Serie A; CA_SA: Counterattacks in Italian Serie A; PA_SP_TT: Positional Attacks in Spanish La Liga – Top teams; PA_SP_MT: Positional Attacks in Spanish La Liga – Intermediate teams; PA_SP_BT: Positional Attacks in Spanish La Liga – Bottom Teams; PA_PL_TT: Positional Attacks in English Premier League – Top teams; PA_PL_MT: Positional Attacks in English Premier League – Intermediate teams; PA_PL_BT: Positional Attacks in English Premier League – Bottom Teams; PA_SA_TT: Positional Attacks in Italian Serie A – Top teams; PA_SA_MT: Positional Attacks in Italian Serie A – Intermediate teams; PA_SA_BT: Positional Attacks in Italian Serie A – Bottom Teams; CA_SP_TT: Counterattacks in Spanish La Liga – Top teams; CA_SP_MT: Counterattacks in Spanish La Liga – Intermediate teams; CA_SP_BT: Counterattacks in Spanish La Liga – Bottom Teams; CA_PL_TT: Counterattacks in English Premier League – Top teams; CA_PL_MT: Counterattacks in English Premier League – Intermediate teams; CA_PL_BT: Counterattacks in English Premier League – Bottom Teams; CA_SA_TT: Counterattacks in Italian Serie A – Top teams; CA_SA_MT: Counterattacks in Italian Serie A – Intermediate teams; CA_SA_BT: Counterattacks in Italian Serie A – Bottom Teams

Post hoc tests identified specific differences among leagues, team levels, and types of attacks. For positional attacks in La Liga, top teams were significantly more efficient than intermediate teams (p < 0.001) and bottom teams (p < 0.001). Intermediate teams were more efficient than bottom teams (p = 0.050). In the Premier League, top teams were significantly more efficient than intermediate teams (p < 0.001) and bottom teams (p < 0.001). Intermediate teams showed higher efficiency than bottom teams (p = 0.007). In Serie A, top teams exhibited significantly higher efficiency than intermediate teams (p < 0.001) and bottom teams (p < 0.001). Intermediate teams were more efficient than bottom teams (p = 0.107).

Regarding counterattacks in La Liga, top teams were significantly more efficient than intermediate teams (p < 0.001) and bottom teams (p < 0.001). However, intermediate teams were not more efficient than bottom teams (p = 0.235). In the Premier League, top teams had significantly higher efficiency compared to intermediate teams (p < 0.001) and bottom teams (p < 0.001). Intermediate teams were more efficient than bottom teams (p = 0.047). In Serie A, top teams showed higher efficiency than intermediate teams (p = 0.005) and bottom teams (p < 0.001). Intermediate teams were more efficient than bottom teams (p = 0.003).

Furthermore, six models were generated in the multiple regression analysis to identify factors influencing the efficiency of positional attacks and counterattacks across the three European soccer leagues analyzed: La Liga, Premier League, and Serie A (Table 3). In La Liga, two models were developed. In the first model, where the dependent variable was the percentage efficiency for positional attacks, the most significant predictor was the efficiency for attacks through the right flank. This was followed by attacks with shots on the left flank and attacks with shots in the center, both of which were also significant. The efficiency for attacks through the central zone was next, followed by the efficiency for attacks through the left flank. The variables counterattacks and attacks on the left flank showed some impact, but were less significant. The most negatively influential factor was counterattacks with a shot, indicating an inverse relationship with the efficiency of positional attacks. In the second model, the percentage of efficiency for counterattacks was the dependent variable. The most significant predictor was attacks with shots in the center, followed by attacks with shots on the left flank and attacks with shots on the right flank, all of which positively correlated with counterattack efficiency. Positional attacks also had a positive contribution. The efficiency for attacks through the left flank and efficiency for attacks through the right flank were important, though less so than the shot-related variables. Accurate passes had a smaller positive effect, while attacks in the center was the most significant negative predictor.

**Table 3:** Stepwise multilinear regression analysis of the association between the effectiveness of positional attacks and counterattacks.

Type of the attack and league	R^2^	*p*-value (model)	Dependent Variable	Independent Variables	Standardized Coefficient (β)	*p*-value (variable)
PA-SP	0.978	<0.001	Percentage of efficiency for positional attacks	Efficiency for Attacks through the Right Flank (Percentage)	0.431	<0.001
				Attacks with Shots on the Left Flank	0.384	0.001
				Attacks with Shots in the Center	0.267	<0.001
				Efficiency for Attacks through the Central Zone (Percentage)	0.182	<0.001
				Efficiency for Attacks through the Left Flank (Percentage)	0.168	0.07
				Counterattacks	0.084	0.004
				Attacks on the Left Flank	−0.134	0.011
				Counterattacks with a Shot	−0.335	<0.001
CA-SP	0.981	0.012	Percentage of efficiency for counterattacks	Attacks with Shots in the Center	1.347	<0.001
				Attacks with Shots on the Left Flank	0.573	<0.001
				Attacks with Shots on the Right Flank	0.561	<0.001
				Positional Attacks	0.536	<0.001
				Efficiency for Attacks through the Left Flank (Percentage)	0.384	<0.001
				Efficiency for Attacks through the Right Flank (Percentage)	0.347	0.001
				Accurate Key Passes	0.13	0.004
Attacks in the Center	−0.32	<0.001
Quantity of Ball Possessions	−0.438	<0.001
Positional Attacks with Shots	−2.188	<0.001
PA-PL	0.967	<0.001	Percentage of efficiency for positional attacks	Efficiency for Attacks through the Right Flank (Percentage)	0.36	<0.001
				Efficiency for Attacks through the Central Zone (Percentage)	0.352	<0.001
				Efficiency for Attacks through the Left Flank (Percentage)	0.301	<0.001
				Percentage of Accurate Passes	0.192	<0.001
				Counterattacks	0.156	<0.001
				Shots	0.154	<0.001
				Attacks on the Right Flank	−0.067	<0.001
				Counterattacks with a Shot	−0.257	<0.001
CA-PL	0.977	<0.001	Percentage of efficiency for counterattacks	Efficiency for Attacks through the Right Flank (Percentage)	0.941	<0.001
				Positional Attacks	0.735	<0.001
				Attacks with Shots in the Center	0.734	<0.001
				Efficiency for Attacks through the Left Flank (Percentage)	0.702	<0.001
				Efficiency for Attacks through the Central Zone (Percentage)	0.571	<0.001
				Attacks with Shots on the Left Flank	0.281	0.005
Quantity of Ball Possessions	−0.307	<0.001
Positional Attacks with Shots	−2.583	<0.001
PA-SA	0.981	0.001	Percentage of efficiency for positional attacks	Efficiency for Attacks through the Right Flank (Percentage)	0.396	<0.001
				Efficiency for Attacks through the Left Flank (Percentage)	0.34	<0.001
				Attacks with Shots in the Center	0.329	<0.001
				Efficiency for Attacks through the Central Zone (Percentage)	0.181	<0.001
				Shots	0.125	0.007
Accurate Passes	−0.087	0.001
Counter-attacks with a Shot	−0.253	<0.001
CA-SA	0.976	<0.001	Percentage of efficiency for counterattacks	Efficiency for Attacks through the Central Zone (Percentage)	0.795	<0.001
				Shots	0.774	<0.001
				Efficiency for Attacks through the Left Flank (Percentage)	0.649	<0.001
				Efficiency for Attacks through the Right Flank (Percentage)	0.523	<0.001
				Passes	0.495	<0.001
				Positional Attacks	0.44	<0.001
				Entrances to the Final Third	−0.421	<0.001
				Positional Attacks with Shots	−1.963	<0.001

Legend: PA-SP: Efficiency of positional attacks in La Liga; CA-SP: Efficiency of counterattacks in La Liga; PA-PL: Efficiency of positional attacks in the Premier League; CA-PL: Efficiency of counterattacks in the Premier League; PA-SA: Efficiency of positional attacks in Serie A; CA-SA: Efficiency of counterattacks in Serie A; R^2^: Coefficient of determination; p-value (model): Model significance level; β: Standardized coefficient; p-value (variable): Significance level of each independent variable

For the Premier League, two models were also generated. In the first model, where the percentage efficiency for positional attacks was the dependent variable, the strongest predictor was the efficiency for attacks through the right flank. The efficiency for attacks through the central zone and the efficiency for attacks through the left flank followed this. The model also indicated a positive influence from the percentage of accurate passes. Variables such as counterattacks and shots had a lesser impact. Attacks on the right flank and counterattacks with a shot had weaker, yet notable, effects on positional attack efficiency. In the second model, the percentage of counterattack efficiency served as the dependent variable. The most significant predictor was efficiency for attacks through the right flank, followed by positional attacks, attacks with shots in the center, and efficiency for attacks through the left flank, all of which positively influenced counterattack efficiency. Efficiency for attacks through the central zone also played a role, though less so compared to the flank-related variables. Attacks with shots on the left flank had a moderate positive effect, and quantity of ball possessions showed a smaller positive influence. Positional attacks with shots had the least impact among the variables.

In Serie A, two models were developed. In the first model, where the percentage efficiency for positional attacks was the dependent variable, the most significant predictor was Efficiency for Attacks through the Right Flank, followed by efficiency for attacks through the left flank. Attacks with shots in the center was another influential factor, followed by efficiency for attacks through the central zone. Shots had a positive effect, while accurate passes had a smaller negative effect. The most significant negative predictor was counterattacks with a shot. In the second model, counterattack percentage efficiency was the dependent variable. The most significant predictor was Efficiency for Attacks through the Central Zone, followed by shots, efficiency for attacks through the left flank, efficiency for attacks through the right flank, accurate passes, and positional attacks, all positively impacting counterattack efficiency. Entrances to the final third was a significant negative predictor, and positional attacks with shots had the most substantial negative impact on counterattack efficiency.

## Discussion

This study aimed to compare the effectiveness of positional attacks and counterattacks across the top three soccer leagues worldwide (i.e., La Liga, Premier League, and Serie A), considering teams from different standings, and to identify factors influencing both attack types. The results revealed that counterattacks were more efficient than positional attacks in all three leagues. Positional attacks and counterattacks were more effective in Serie A compared to the Premier League, while the Premier League showed greater efficiency than La Liga. In all three leagues, teams at the top of the table demonstrated greater effectiveness in both types of attacks than intermediate teams, and intermediate teams were more effective than those at the bottom. Additionally, positional attack efficiency was mainly associated with attacks through the right flank, left flank, and central zone, while counterattacks with a shot were a negative predictor of positional attack efficiency. Similarly, counterattack efficiency was primarily predicted by attacks with shots in the center, attacks from the flanks, and efficiency for attacks through the flanks and central zone, while the quantity of ball possession and positional attacks with shots were negative predictors of counterattack efficiency.

When analyzing the comparative effectiveness of positional attacks versus counterattacks, the reasons why counterattacks are more efficient can be attributed to several factors intrinsic to the nature of counterattacks. Unlike positional attacks which demand a complex buildup involving multiple passes and intricate movements, counterattacks capitalize on the immediate transition from defense to offense (Hewitt et al., 2016). In consideration of a whole match, this rapid response requires fewer resources (e.g., accelerations, decelerations, high-intensity, sprint distances) and tends to be executed with greater speed and high intensity at the moment of attack compared to positional attacks (Forcher et al., 2023). Because of this, counter-attacks offer the opportunity to exploit open spaces when the opposing defense is often disorganised and unprepared, as it is in transition from an offensive position (Forcher et al., 2023; Hewitt et al., 2016). This disorganization leaves gaps that the attacking team can swiftly exploit. The necessity for defenders to rapidly retreat and reorganize often results in a defensive unit that is less cohesive and more susceptible to quick, decisive attacks (Li and Zhao, 2021). Moreover, the speed of counterattacks creates significant challenges for defenders. The high velocity with which counterattacks are executed and the element of surprise hamper the defense's ability to respond effectively. Defenders are often forced into a reactive position, trying to cover spaces and hinder attackers' advance. This increased difficulty in defensive coverage further enhances the effectiveness of counterattacks compared to the more deliberate and methodical approach of positional attacks. Despite these results, previous research (Forcher et al., 2023) indicated that the influence of the style of play (counterattack vs. positional attack) was small in terms of success. In this line, it was emphasized that chance, rather than the style of play, determined a team's success (Brechot and Flepp, 2020). On the contrary, others, such as Yi et al. (2019), concluded that a positional attack was the best option to obtain the best results. Because the samples of all these studies are disparate, those results suggest that the teams' competition and quality should be considered when comparing the effectiveness of positional attacks vs. counterattacks (Kempe et al., 2014).

Considering the competition analysed, our results showed differences among all of them. Firstly, Serie A showed superior effectiveness in both positional attacks and counterattacks, which could be attributed to the high tactical discipline of the teams participating in this league (Sarmento et al., 2013). This tactical rigor may enable teams to exploit defensive mistakes by their opponents more effectively. Additionally, the defensive efficiency in Serie A could facilitate ball recovery and the initiation of counterattacks under advantageous conditions, ultimately leading to successful shots at the goal. Secondly, the Premier League demonstrated strong performance in both types of attacks, although its effectiveness was slightly lower than that of Serie A (Sarmento et al., 2013). This could be due to the Premier League's faster-paced and physically demanding nature. The higher tempo and a more aggressive style of play may lead to less tactically sophisticated positional attacks and counterattacks than those observed in Serie A (Mitrotasios et al., 2019; Prieto-González et al., 2024). The lower effectiveness of La Liga in both positional attacks and counterattacks, compared to Serie A and the Premier League, might indicate a different tactical emphasis or less effective implementation of these strategies. Finally, La Liga is often characterized by a focus on technical skill and possession-based play (Prieto-González et al., 2024), which does not always translate into the same level of efficiency for positional attacks or counterattacks seen in other leagues. Despite this, it seems that the percentage of possession is not a determining factor in predicting match success. In La Liga, teams may prioritize passing precision and control over speed of play, resulting in greater possession and fewer turnovers, but potentially reduced ability to surprise opponents, and consequently, lower effectiveness in both types of attacks.

Considering the level of the team in each of the leagues analysed, it could be observed that the top teams were more effective than the middle teams and that the middle teams were more effective than the bottom teams. These results can be attributed to the superior physical, technical, tactical, and strategic capabilities of players in the top teams (Collet, 2013; Kuvvetli and Çilengiroğlu, 2024; Vogelbein et al., 2014). Considering the results of the multiple regression analysis, in all three leagues, the efficiency for attacks through the right flank emerged as the most critical predictor of positional attack efficiency, reflecting a consistent tactical preference across different soccer cultures. Effective use of the right flank likely created more successful attacking opportunities, leading to higher overall efficiency. Meanwhile, the efficiency of the left flank and the center remained relevant in all leagues, although its significance varied. This variation might be due to different team strengths, play styles, or defensive schemes employed by opponents. In agreement with these results, Taylor et al. (2005) indicated that in La Liga, the offensive efficiency was greater on the right side because the weak defensive zone was the left. Meanwhile, other studies found that central attacks were the most decisive in La Liga compared to flank attacks (Guimarães et al., 2022; Prieto-González et al., 2024). In addition, it should be noted that the zone in which the ball is recovered marks the beginning of the offensive action and, therefore, influences the success of a team. In this regard, Barreira et al. (2014) observed that during the 2010 FIFA World Cup, the winning team recovered most of the possessions and initiated the attack mainly on the right side. However, Jamil (2019) found that in the Premier League from 2015–2016 to 2017–2018, possession recoveries on the left side of the field of play led to greater productivity in front of the goal. In addition, players who played on the left side of the pitch were more productive. In contrast, in La Liga, most of the attacks originated in the center (Papadopoulos et al., 2021).

Additionally, the negative association between shots from counterattacks and positional attack efficiency observed in La Liga and Serie A highlights an intriguing dynamic. This suggests that teams emphasizing counterattacks might not be as effective in creating high-quality shots or maintaining efficient positional play. Quick, opportunistic counterattacks often lack the structured approach needed for high-efficiency positional attacks, potentially leading to less effective shots and a decrease in overall efficiency when these opportunities are relied upon excessively. These findings reinforce the idea that positional attacks could be the most effective in La Liga and Serie A (Prieto-González et al., 2024; Sarmento et al., 2013). Furthermore, in order to perform an effective positional attack, successful teams in these leagues must have high passing accuracy (Brito Souza et al., 2019; Plakias et al., 2022; Prieto-González et al., 2024). Meanwhile, in the Premier League, counterattacking would be the most successful attack (Harrop and Nevill, 2014; Kite and Nevill, 2017). Contrary to the present study results’ and previous findings, Sarmento et al. (2018) noted that counterattacking was 40% more effective than positional attacking in La Liga, Serie A, and Premier League. These differences may be because those authors only analyzed three teams during the 2013/2014 and 2014/2015 seasons, thus the sample was insufficient to define the predominant playing style of the entire league.

The regression analysis also highlighted some key differences among the leagues. In line with previous studies, and even though direct play was predominant (Harrop and Nevill, 2014; Kite and Nevill, 2017), in the Premier League, the percentage of accurate passes emerged as a notably important factor, underscoring the critical role of passing precision for achieving high efficiency in positional attacks, in contrast to La Liga and Serie A. Serie A showed a positive impact of shots during positional attacks, which was less pronounced in La Liga and the Premier League, indicating a stronger emphasis on converting shooting opportunities into successful attacks. Additionally, in Serie A, the effect of accurate passes was negative, differing from the positive influence observed in the Premier League and the lesser impact in La Liga. These differences reflect how each league's tactical and strategic preferences shape the factors influencing the efficiency of positional attacks. In La Liga, the focus remains heavily on efficiency through the right flank, with a notable negative impact of counterattacking shots. The Premier League values precise passing, while Serie A highlights the importance of effective shooting and balances this with a critical view on pass accuracy. Despite these differences, existing literature has shown that passing accuracy and shooting efficiency are major success factors in soccer (Harrop and Nevill, 2014; Kite and Nevill, 2017; Lago-Ballesteros and Lago-Peñas, 2010; Prieto-González et al., 2024).

Moreover, based on the regression analysis, in all three leagues, efficiency for counterattacks was notably influenced by the effectiveness of attacking through the center and the efficiency of the flanks. Efficiency for attacks through the central zone consistently emerged as a significant predictor for counterattack efficiency, highlighting the importance of central play across different soccer contexts. Similarly, efficiency in attacks through both the left and right flanks played a crucial role, although its impact varied slightly among leagues. Additionally, the role of shots was universally important in determining counterattack efficiency, emphasizing that creating and converting opportunities was key across all leagues. Consistent with our results, it appears that regardless of the zone in which it occurs, previous studies have indicated that the key factor in a counterattack is the ability to convert the play into an accurate shot at the goal (Prieto-González et al., 2024) and that teams with less offensive success show lower accuracy in shooting at the goal (Lago-Ballesteros and Lago-Peñas, 2010). However, the presence of entrances to the final third also revealed a pattern; in La Liga and Serie A, an increase in these entrances correlated with lower counterattack efficiency, suggesting a complex relationship between advanced positioning and successful counterattacking. Overall, these similarities underscored the commonality in how central and flank efficiency, as well as shot effectiveness (Lago-Ballesteros and Lago-Peñas, 2010; Prieto-González et al., 2024), shaped counterattack performance across different soccer leagues.

The regression analysis also revealed differences among the leagues regarding counterattack efficiency. These differences illustrate how each league's unique tactical and strategic preferences influence the factors affecting counterattack efficiency. La Liga focuses on flank effectiveness, the Premier League values passing precision, and Serie A emphasizes shot quality and the balance of positioning (Sarmento et al., 2013). The offensive play of fullbacks can explain the effectiveness of counterattacks from the flanks in La Liga. Players occupying this position perform the highest number of crosses, passes, and ball touches in the offensive (Andrzejewski et al., 2015), defensive, and middle zones (Li and Zhao, 2021). In addition, they tend to press the opponent's flanks, which leaves the lateral defensive zone empty and improves the success rate of the opponent's counterattack from the flanks (Li and Zhao, 2021). Previous studies have indicated that these differences in the playing style among each league are influenced by combination of historical, cultural, social, technical and physical factors (Li and Zhao, 2021; Mitrotasios et al., 2019; Sarmento et al., 2013).

This study has some limitations that should be acknowledged. It focuses exclusively on the top three European leagues—La Liga, the Premier League, and Serie A—which may limit the generalizability of the findings to other leagues or competitions. Additionally, the research spanned five seasons but did not account for potential dynamic changes within seasons or the impact of specific match contexts, such as critical matches, weather conditions, or player injuries. To address these limitations, future studies might include data from additional prominent leagues worldwide to improve the generalizability and robustness of the findings. Analyzing data from high-profile competitions such as the Champions League or the World Cup could also provide valuable insights. Moreover, incorporating analyses that consider match contexts and situational variables, such as home versus away games, player fatigue, and the importance of the match, would offer a deeper understanding of attack effectiveness.

## Conclusions

Counterattacks consistently proved more effective than positional attacks in La Liga, the Premier League, and Serie A. Serie A led in effectiveness for both attack types, with the Premier League following and La Liga being the least effective. Top teams performed better in both attack types compared to those in the intermediate and bottom positions. The effectiveness of attacks varied significantly based on the specific factors influencing each league, such as the Premier League’s emphasis on accurate passing, Serie A’s focus on effective shooting, and La Liga’s reliance on right flank attacks. These factors, as well as the role of central and flank efficiency, demonstrated that tactical precision and situational execution are crucial for optimizing attack effectiveness.

## Practical Implications

Based on the study's findings, coaches can adapt their strategies to the specific tactical trends observed in each league. For example, in leagues such as Serie A, where defensive stability is often prioritized, training players to quickly transition from defense to attack could prove essential. In contrast, leagues like La Liga, emphasising possession and positional play, may benefit from creating and maintaining offensive structures. Additionally, the study highlights the importance of exploiting wing play. Therefore, coaches should focus on developing wingers' ability to deliver precise crosses and make impactful runs.

Understanding these league-specific tendencies can help refine individual player roles and team strategies, ultimately enhancing overall performance.
